# Remarkably Few Sputum Cultures from People with Parkinson's Disease during Hospital In-Patient Admission

**DOI:** 10.1155/2015/378967

**Published:** 2015-02-05

**Authors:** Richard W. Walker, Joel English, Grace Tan, Annette Fisher, William K. Gray

**Affiliations:** ^1^Department of Medicine, North Tyneside General Hospital, Northumbria Healthcare NHS Foundation Trust, North Shields, Tyne and Wear NE29 8NH, UK; ^2^Institute of Health and Society, Newcastle University, Newcastle upon Tyne NE2 4AX, UK; ^3^The Medical School, Newcastle University, Newcastle upon Tyne NE2 4HH, UK; ^4^Hospital Jitra, Jalan Changlun, 06000 Jitra, Kedah, Malaysia

## Abstract

Although respiratory tract infections can be a common complication in people with Parkinson's disease (PD), there is little published data on the nature of such infections in this patient group. We wished to investigate whether sputum samples were being taken from PD patients in order to establish whether an infection was present and if so which bacteria were responsible for the infection. We recorded the number of positive sputum samples taken from admission to North Tyneside General Hospital in North-East England across a ten-year period from June 2001 to June 2011. Of 643 in-patient episodes involving people with PD, positive sputum samples were recorded for only 12 episodes (1.9%) in eight patients. All patients were in early stage disease. In all admissions to the NHS Trust running the hospital, there were 23,069 sputum cultures from 1,056,693 in-patient episodes (2.2%). Our findings may reflect the difficultly of expectorating in many people with PD, particularly in late-stage disease. Since people with PD are especially vulnerable to respiratory tract infections, clinicians need to ensure that, where possible, a sputum sample is obtained from people with PD when clinically indicated.

## 1. Introduction

Respiratory tract infections can be a common complication in people with Parkinson's disease (PD). Indeed, 45% of all death certificates for people who had PD have pneumonia listed as a cause of death, a greater proportion than that seen in the general population [1]. The reasons for this are likely to be multifactorial, with reduced mobility during late-stage disease, swallowing difficulty, and impaired cough reflex likely to play major roles [2, 3]. Respiratory dysfunction has also been linked to swallowing problems in people with PD [4]. Identifying the nature of respiratory tract infections allows optimal patient care. As part of an audit within a UK National Health Service (NHS) general hospital, we wished to investigate how many sputum cultures were recorded for people with PD. We hoped that this would act as a guide as to how well such infections are monitored during in-patient hospital visits.

## 2. Methods

The study was registered with Northumbria Healthcare NHS Foundation Trust's research department and approval granted from the Caldicott Guardian. Ethical approval was not required for this retrospective audit of patient's notes.

Northumbria Healthcare NHS Foundation Trust provides acute care for a population of around 500,000 people in northeast England.

The trust runs three general hospitals including North Tyneside General Hospital which covers a population of around 200,000 people. The trust's PD service cares for around 700 people with PD within its catchment area. The vast majority of people with PD in the catchment area are served by the trust [5, 6]. Microbiology archives and a register of people with PD cared for by the PD team at North Tyneside General Hospital were used to locate PD patients with positive sputum cultures during the ten-year period from June 2001 to June 2011. PD diagnosis was according to the UK Brain Bank Criteria. Organism was noted and the case notes were reviewed for clinical details.

Trustwide data were also obtained regarding the number of positive sputum cultures within the entire trust and the total number of in-patient episodes (elective, nonelective, and day cases) during the period.

## 3. Results

Of 643 in-patient episodes at North Tyneside General Hospital involving people with PD during the ten-year study period, only 12 (1.9%) positive sputum samples (in eight patients) were recorded. Within the trust, there were 1,056,693 in-patient episodes over this period and 23,069 (2.2%) positive sputum cultures.

One patient had three positive samples and two patients had two positive samples during a single in-patient stay. Seven of the eight patients were male and all were in early stage disease at the time of their stay (two having Hoehn and Yahr stage I, three having Hoehn and Yahr stage II, and three having Hoehn and Yahr stage III).

Eight different types of organisms were detected, including* S. aureus* (*n* = 3),* H. influenzae* (*n* = 3), coliform (*n* = 2),* C. albicans* (*n* = 2),* S. pneumoniae* (*n* = 2),* E. coli* (*n* = 1),* Haemophilus* sp. (*n* = 1), and* M. catarrhalis* (*n* = 1). Two patients had multiple organisms.

## 4. Discussion

Pneumonia is a common complication in people with PD, particularly in later-stage disease. In a study from 2005 by members of our team, pneumonia was responsible for 11.4% of all emergency hospital admissions and was the second most common cause of admission after falls [7]. A more recent UK-wide study of elective and nonelective hospital admissions supports these findings [8]. The authors found pneumonia to be the leading cause of admission in people with PD during a four-year period from 2009 to 2013, with 13.5% of 232,905 emergency PD admissions due to pneumonia. In the general population aged 35 years and over, pneumonia was responsible for only 7.0% of admissions.

Given the fact that pneumonia appears to be more common in people with PD than the general population, there were remarkably few positive sputum samples during the ten-year period of our study [7]. Indeed, since pneumonia is generally more common in older age, the fact that a greater percentage of all admissions across all age groups had a sputum sample taken than did people with PD is notable.

Further analysis of these data is hampered by the small number of positive sputum cultures found. A larger, prospective study is merited. Interestingly, all of the samples were in people with early stage PD; this may reflect the difficulty of those in later-stage disease to expectorate and may be the main reason why so few positive cultures were found. Indeed, those who had positive sputum cultures were of much earlier disease stage than typically seen for hospital in-patient admissions in people with PD, suggesting that this may well be the case [7, 9]. Chest infection is reported as one of the most common reasons for hospital admission of people with PD [7, 9]. Swallowing difficultly, a well-recognised symptom of PD that increases in later stages, is likely to be partly responsible for this [2, 4].

Although obtaining a sputum sample from someone in mid- to late-stage disease may be difficult, all patients admitted to hospital with respiratory symptoms should, where physically possible, have a sputum sample taken to ensure appropriate and effective treatment. Future prospective research should investigate if enough is being done to obtain such samples and suggest what measures can be put in place to ensure that, where possible, samples are obtained. Greater involvement of physiotherapists in the care of hospitalised PD patients may be a useful starting point for any intervention and is recommended in recent European guidelines on the role of physiotherapy for people with PD [10].
